# Life expectancy with and without cognitive impairment by diabetes status among older Americans

**DOI:** 10.1371/journal.pone.0190488

**Published:** 2017-12-29

**Authors:** Carlos Díaz-Venegas, Daniel C. Schneider, Mikko Myrskylä, Neil K. Mehta

**Affiliations:** 1 Max Planck Institute for Demographic Research, Rostock, Germany; 2 Department of Social Policy, London School of Economics, London, England; 3 Department of Social Research, University of Helsinki, Helsinki, Finland; 4 School of Public Health, University of Michigan, Ann Arbor, Michigan, United States of America; Nathan S Kline Institute, UNITED STATES

## Abstract

Diabetes affects mortality and cognitive functioning. It is not known how diabetes influences life expectancy (LE) with and without cognitive impairment. We seek to examine age at onset of cognitive impairment and life expectancy (LE) with and without cognitive impairment by diabetes status among middle- and older-aged Americans. Data come from the U.S. Health and Retirement Study 2000–2012 linked to the National Death Index (N = 13,687). We use multinomial regression models stratified by gender and controlling for age, education and race/ethnicity to estimate diabetes-status specific transition probabilities, then use a Markov chain matrix population model to calculate age at onset of cognitive impairment and LE with and without cognitive impairment by diabetes status at age 50. LE at age 50 was 27.6 (men) and 32.1 (women). From age 50, those with diabetes had a first incidence of cognitive impairment 3 (men) and 4 (women) years earlier than those without. Diabetes reduced total LE by 5–7 years and cognitively healthy LE by 4–6 years. Those with diabetes lived one year less in a cognitively impaired state than those without. Over 80% of the lower LE associated with diabetes is attributable to the loss of cognitively-healthy years. Those with diabetes have a shorter LE with cognitive impairment because of higher mortality. In analyses by educational attainment, education was strongly protective of cognitive health, yet diabetes was associated with lower age of cognitive impairment onset and fewer cognitive healthy years lived in all educational groups. The excess mortality because of diabetes may be decreasing. If the mortality decline is not coupled with a comparable decline in the risk of cognitive impairment, the population level burden of impaired cognition may grow larger.

## Introduction

Diabetes is a disabling and lethal condition associated with a reduction in life expectancy of nearly a decade [1–3]. According to the American Diabetes Association, in 2012, around 9.3% of the U.S. population had diabetes, representing over 29 million individuals [4].

In addition to substantially raising the risk of death, diabetes is an important risk factor for cognitive impairment. A recent estimate suggests that diabetes is associated with a 40% increase in the odds of dementia among older Americans [5]. The association between diabetes and cognitive impairment was noted by Miles and Root in 1922 [6]. Nearly a century later, the precise physiologic pathways linking the two conditions remain largely undetermined. Hypothesized pathways include those related to neuronal glucose processing, cerebrovascular complications, and frequent episodic hypoglycemia [7–9]. Individuals with diabetes are also more likely to have comorbid cardiovascular disease, which is itself predictive of cognitive decline through cerebrovascular events and other pathways [10].

Given that cognitive impairment is a major cause of loss of independence [11], presents a barrier to medication adherence [12], and results in extremely high care costs [13], policies aimed at improving outcomes among those with diabetes should be informed by the level of cognitive impairment in this population. It would be expected that those with diabetes experience an earlier age at onset of cognitive impairment compared to those without diabetes. However, despite the documented links between diabetes and cognitive function, we do not know how much earlier that age is. Furthermore, we do not know how long those with diabetes are expected to live in a cognitively impaired state. It may be that those with diabetes experience an earlier onset of cognitive impairment but once that impairment develops they go on to experience a shorter life compared to those without diabetes.

Using a nationally representative longitudinal sample of Americans aged 50 years and older that contains high quality indicators of cognitive functioning, we sought to estimate the age at onset of cognitive impairment and life expectancy with and without cognitive impairment by diabetes status. We analyze the results by sex as well as by education, which is a key socioeconomic factor that has been known to affect the risks of developing diabetes and cognitive impairment. In the context of our analyses, a higher educational attainment might bring access to higher quality of health care (including preventative care) and also the possibility of engaging in healthy lifestyle behaviors [14], all of which may protect the highly educated from the negative cognitive impacts of diabetes. A key strength of our study is reliance on longitudinal data to ascertain the age-specific incidence of cognitive impairment.

## Data and methods

### Sample

The Health and Retirement Study (HRS) is a nationally representative panel of Americans over age 50 that contains information on health, housing, disability, and cognition, as well as a wide set of socio-demographic variables. Baseline data collection for the HRS was in 1992 and included a combination of in-person and telephone interviews of individuals born between 1931 and 1941 (82% response rate). Follow-up interviews were conducted every 2 years, and new birth cohorts were added approximately every six years [15]. Since 1998, the HRS has been nationally representative of non-institutionalized American adults ages 50 and over. Individuals who entered nursing homes or who were otherwise institutionalized were retained in the study. The Health and Retirement Study has the approval granted by the appropriate IRB and Ethics Committee. However, our study does not require a similar consent as secondary data was analyzed anonymously. We included all individuals who were aged 50–74 years in 2000 and followed them until death or censoring which was at the date of the 2012 survey or earlier if the person was lost to follow-up. The total sample size was 13,687 individuals who contributed 136,367 person-years of follow-up over the years 2000–2012.

Attrition plays a role in every longitudinal study. In our case, 2.3% of the respondents were lost to follow-up and dropped from the sample at some point during the 12 years we have considered for this study. Finally, 13.8% of the interview responses in the cognition variable were missing. These respondents were on average 5.5 years older, more likely to not have attended college, more likely to be female, and had a slightly higher prevalence of diabetes than the respondents included in the sample. If the missingness is negatively correlated with health, then it is possible that our estimates of diabetes prevalence and cognitive burden are conservative lower bounds.

### Measures

Diabetes status was ascertained based on the variable “has [the respondent] ever been diagnosed with diabetes at interview”. We created a dichotomous variable indicating diagnosed diabetes status based on the response. Once the respondent has reported the presence of diabetes, we keep the individual with that status until the end of our study or until death.

Cognitive function was measured based on the responses to the modified version of the Telephone Interview for Cognitive Status (TICS-M) test, which was administered in every wave [16, 17]. The test score ranges from 0 to 27 points and includes four tasks: immediate verbal recall (0–10 points), delayed verbal recall (0–10 points), serial 7’s (0–5 points), and counting backwards (0–2 points) [18]. Following standard protocol, scores of 11 points or less were defined as cognitive impairment [19]. This group includes individuals who have cognitive impairment without dementia (i.e., CIND; with a score of 7–11 points) and those with dementia (with a score of 0–6 points).

We adjusted for educational attainment (less than high school, high school degree or some college, and college graduate) and also race/ethnicity (non-Hispanic white, non-Hispanic black, Hispanic, other non-Hispanic race). Results stratified by educational attainment are also presented.

### Statistical analysis

A person-year file was constructed from the data. Multinomial logistic models stratified by initial cognition state (non-impaired and impaired) were run to estimate transition probabilities across states. The first regression, restricted to instances of a non-impaired initial state, provided transition probabilities for "remaining non-impaired", "becoming impaired", and "dying from a non-impaired state”. The second regression, restricted to instances of an impaired initial state, provided analogous transition probabilities from the state of cognitive impairment.

We controlled for age and age squared, race/ethnicity, and education. We included the binary variable indicating whether the respondent had ever been diagnosed with diabetes at the time of the interview. The above models were estimated for males and females separately.

From the multinomial logistic model results, we predicted age- and gender-specific transition probabilities across six combinations: four combinations among alive states (staying not impaired; staying impaired; impaired to not impaired; not impaired to impaired) and two transitions to death (not impaired to dead; impaired to dead). In these predictions, race/ethnicity and education were set to their overall sample mean and age successively took on values from 50 to 110. We included these transitions because literature recognizes that persons with some degree of cognitive impairment can remain stable and some even revert to normal cognition [20]. In fact, some population-based observational studies have reported between 29% and 55% of respondents with some degree of cognitive impairment have recovered [21].

We then applied the transition probabilities into a Markov chain matrix population model in order to calculate total life expectancy, the expected time spent in cognitively non-impaired and impaired states, and age at first onset of cognitive impairment, all by gender and diabetes status [22]. In some of the analyses we generated three different scenarios. The first, labeled Observed, is based on the actual observed prevalence of diabetes and non-diabetes in the sample. The second, labeled No Diabetes, assumes zero diabetes prevalence in the sample. The third, labeled Full Diabetes, is calculated for a sample in which everyone eventually becomes diabetic before death. In this scenario, the age-specific transition probabilities reflect the transition rates of individuals who by that age have diabetes, and the distribution of diabetes diagnoses by age is the observed distribution among those with diabetes. All the procedures were done using Stata/SE version 14.2 [23].

## Results

Table 1 describes the sample overall and by diabetes status. Prevalence of diabetes is 14%. Mean age is 65 years and 57% were female. Approximately a quarter of the sample has less than high-school education, and a fifth are college graduates. Around 76% are non-Hispanic whites, 14% are non-Hispanic blacks, 8% Hispanics, and 2% are from other racial backgrounds. The mean cognition score was 16 points and 15% of the sample is categorized as being cognitively impaired at study entry (score of 11 points or less). Over the follow-up, 4,247 persons died. Around 30% of the transitions from a non-impaired state to an impaired one or from an impaired state to a non-impaired one happened among respondents with diabetes.

**Table 1:** Characteristics of the sample population at study entry, years 2000–2012.

Characteristics		Total	By Diabetes Status		Test Difference P-Value
			Yes	No	
Has diabetes, %		14.4			
Mean Age, years		65.0	65.9	64.9	0.000
Gender, %	Female	57.0	52.6	57.7	0.000
	Male	43.0	47.4	42.3	0.000
Education, %	Less than High School	23.8	36.0	21.8	0.000
	High School Degree/GED	57.0	50.5	58.0	0.000
	College Graduate	19.2	13.5	20.2	0.000
Race/ Ethnicity, %	Non-Hispanic Whites	76.1	61.9	78.5	0.000
	Non-Hispanic Blacks	13.9	23.6	12.2	0.000
	Other Races	2.0	2.2	2.0	0.530
	Hispanics	8.0	12.3	7.3	0.000
Cognition	Mean Cognition Score, range 0–27	15.9	14.5	16.2	0.000
	-- No Impairment, % (score > 11)	84.6	75.1	86.1	0.000
	-- Cognitive Impairment, % (score < = 11)	15.4	24.9	13.9	0.000
Mortality	Number of Deaths	4,247	1,048	3,199	
	Crude death rate/1000 (deaths/person-years)	31.1	37.1	29.6	0.000
Transitions	From Non-Impaired to Impaired	5,546	1,493	4,053	
	From Impaired to Non-Impaired	3,783	987	2,796	
Sample size	Number of persons	13,687	1,975	11,712	
	Person-Years of Follow-Up	136,367	28,269	108,098	

**Note**: Authors’ own elaboration with data from the Health and Retirement Study. Values shown are unweighted. All values are percentages unless noted. Column percentages were used for gender, educational attainment and race/ethnicity so each segment adds up to 100%.

Table 1 shows considerable differences for those with and without diabetes that highlight the mortality and cognition disadvantages that are associated with diabetes. Those with diabetes are more likely to have less than a high school degree (36% vs. 22%; p ≤ 0.01) and are more likely to be non-Hispanic black (23% vs. 12%; p ≤ 0.01). The average cognition score for those with diabetes is 1.7 points lower and the prevalence of cognitive impairment is more than 10% higher than for those without diabetes (25% vs. 14%; p ≤ 0.01). The crude death rate is 37.1 deaths per 1,000 person-years for individuals with diabetes, which is 25% higher (p ≤ 0.01) than those without diabetes (29.6 deaths per 1,000 person-years).

**Fig 1:**
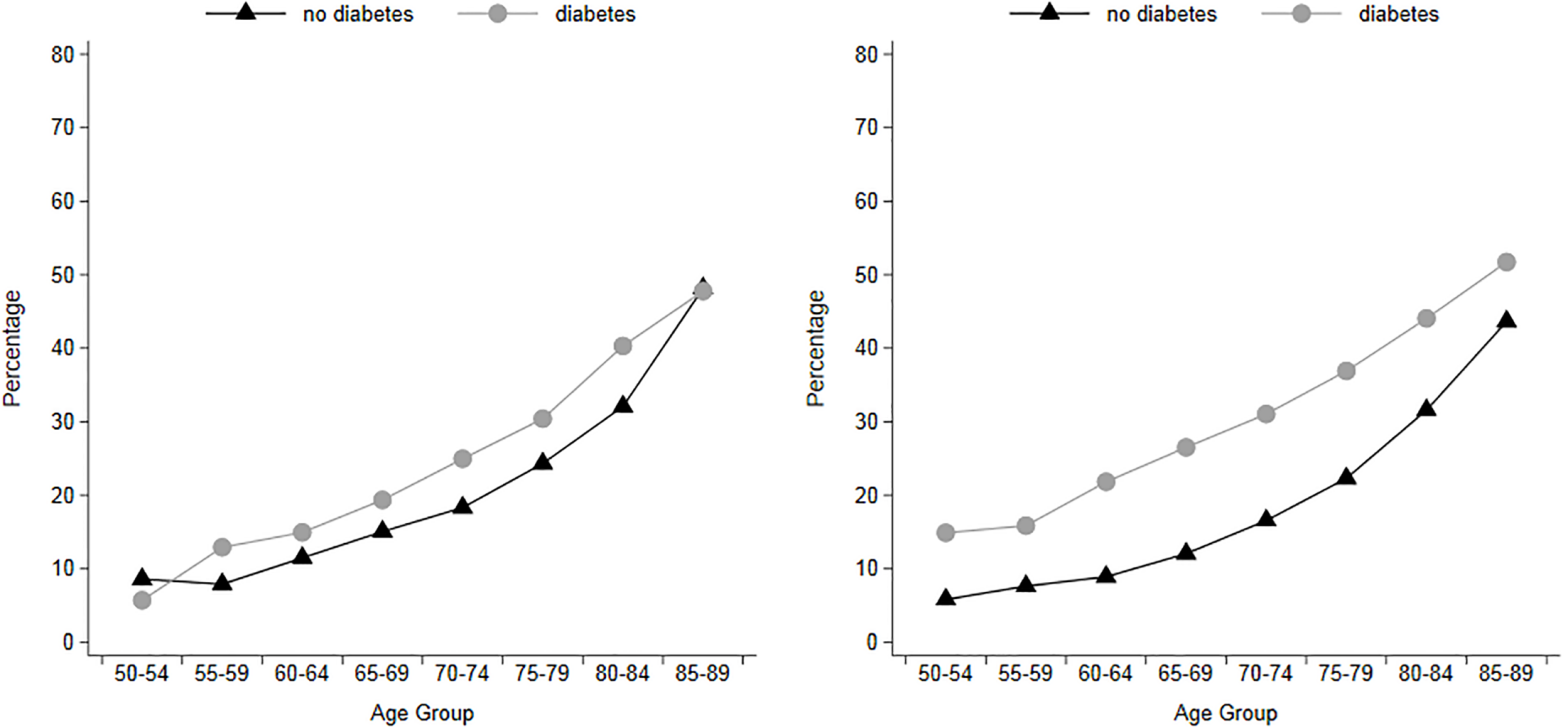
Prevalence of cognitive impairment of older adults in the U.S. for men (left) and women (right) by diabetes status and age groups, 2000–2012. **Note**: Authors’ own elaboration with data from the Health and Retirement Study.

Fig 1 shows the age-specific prevalence of cognitive impairment by gender and diabetes status. For both men and women, and for those with and without diabetes, prevalence of cognitive impairment increases with age, being close to 10% at ages 50–54, and reaching levels that are above 40% by age 85–89. The differences in prevalence of cognitive impairment by diabetes status are more pronounced for women than for men. For both genders and for most ages, those with diabetes have a higher prevalence of cognitive impairment than those without. The exceptions to this rule are men aged 50–54, for whom cognitive impairment is comparatively rare (less than 10%) and men aged 85–89, among whom selective mortality among those with diabetes may influence the prevalence of cognitive impairment.

**Fig 2:**
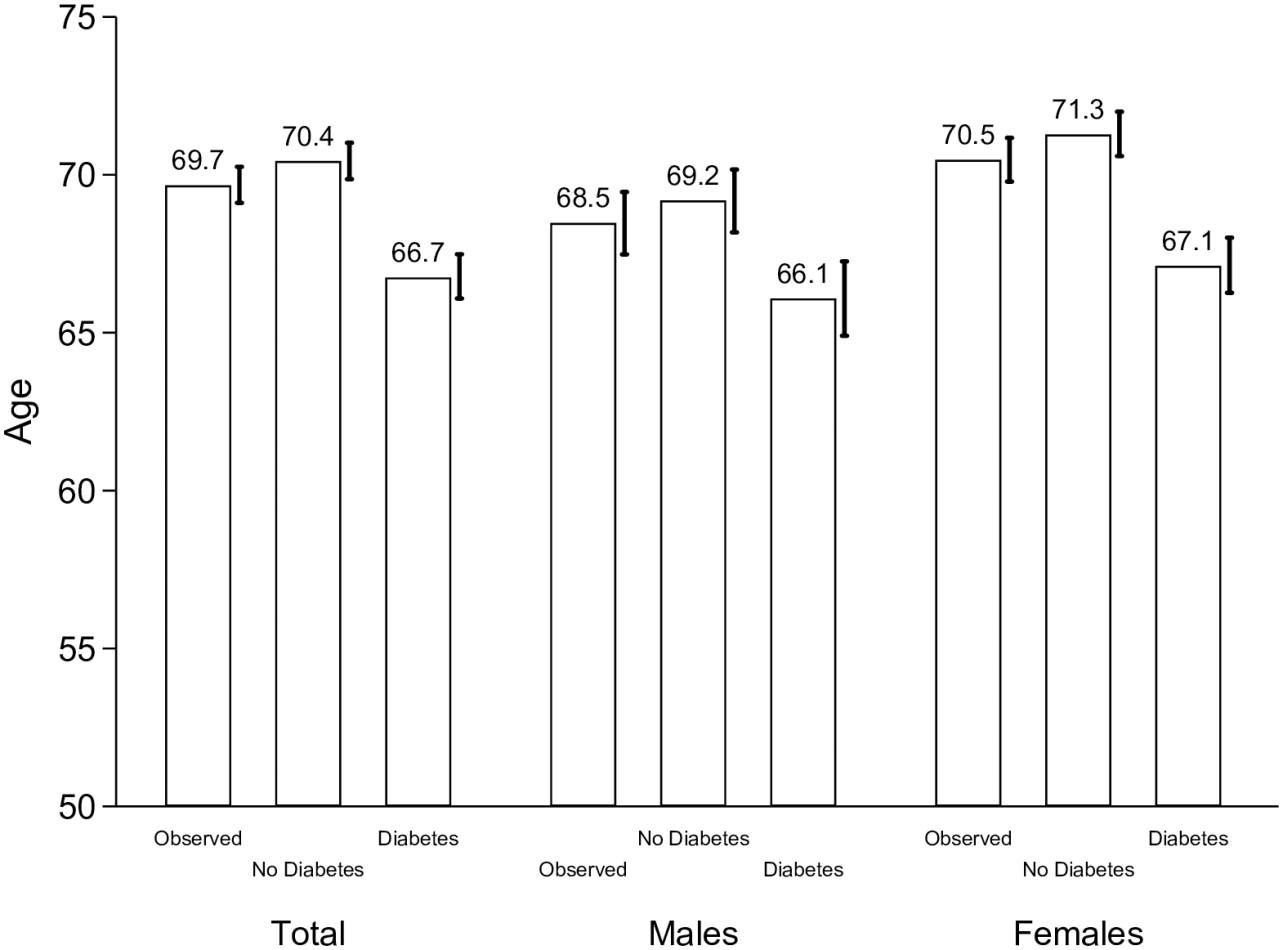
Mean age of first incidence of cognitive impairment by diabetes status, 2000–2012. **Note**: Authors’ own elaboration with data from the Health and Retirement Study. “Observed” refers to the average prevalence of diabetes. “No Diabetes” assigns zero diabetes to the sample population. “Diabetes” assigns full diabetes to the sample population. The 95% confidence intervals, which are based on 1,000 bootstrap replications, are included on the right of each column.

Fig 2 shows the mean age of first incidence of cognitive impairment by gender and diabetes status for individuals who are not cognitively impaired at age 50. For the sample including both men and women, the mean age at first incidence from non-impaired to impaired cognitive function is 69.7 years. Among men, it was 68.5 years and among women it was 70.5. For the No Diabetes scenario, the mean ages at first transition are around 0.7–0.8 years higher than in the Observed scenario: 70.4 years for the entire sample, 69.2 years for men and 71.3 years for women. Compared to the No Diabetes scenario, in the full Diabetes scenario the mean age at first transition is 3.7 years lower for the entire sample (66.7 years), 3.1 years lower for men (66.1 years) and 4.2 years lower for women (67.1 years).

**Fig 3:**
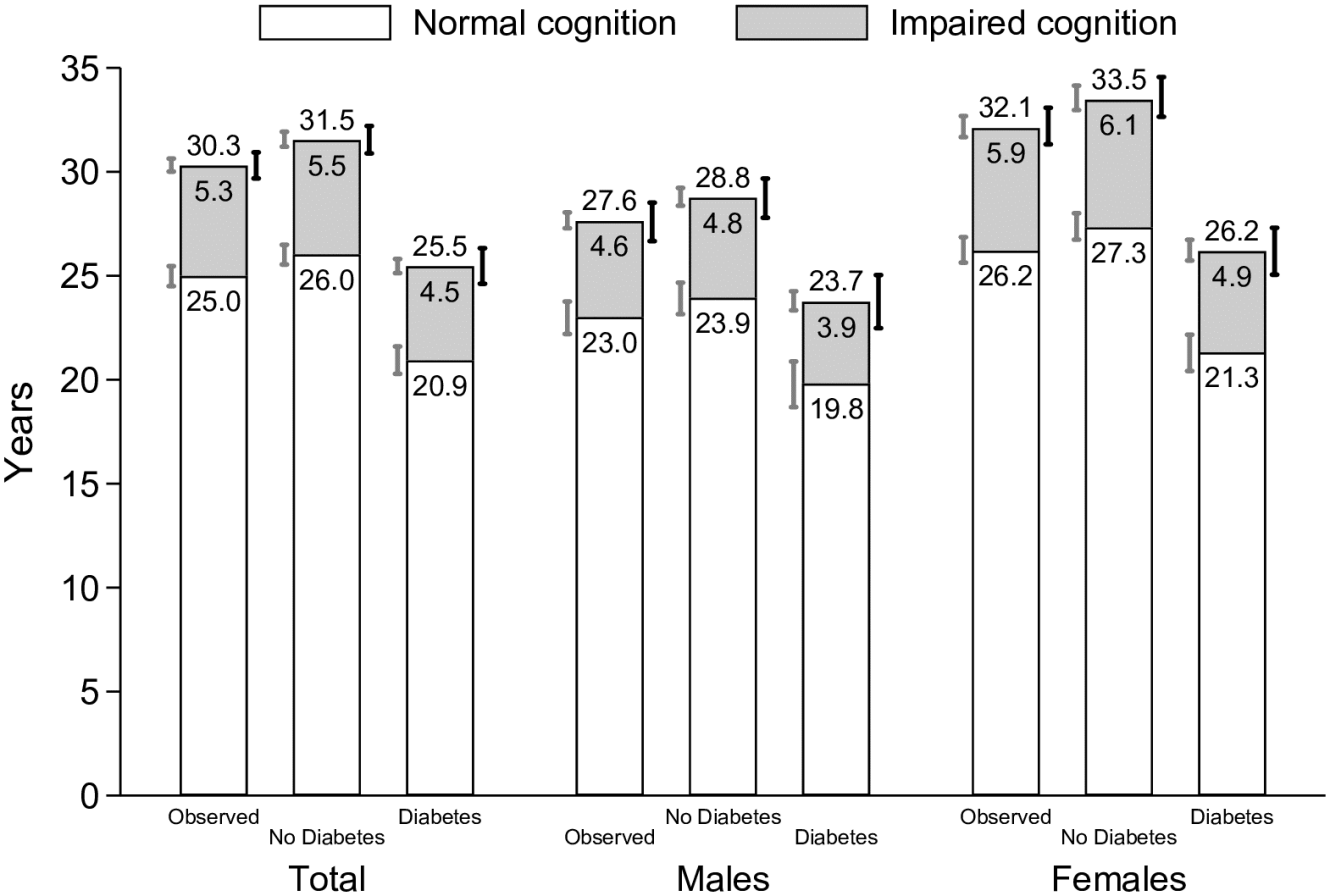
Cognitive impaired and cognitive-impaired free life expectancies at age 50 for older adults in the U.S. by gender and prevalence of diabetes. **Note**: Authors’ own elaboration with data from the Health and Retirement Study. “Observed” refers to the average prevalence of diabetes. “No diabetes” assigns zero diabetes to the sample population. “Diabetes” assigns full diabetes to the sample population. The 95% confidence intervals on the left of each column (gray) refer to the healthy life expectancy (normal cognition) and to the cognitively-impaired life expectancy (impaired cognition). The 95% confidence intervals, which are based on 1,000 bootstrap replications, on the right of each column (black) refer to the total life expectancy. Both non-impaired and cognitively-impaired life expectancies might not add up to the total life expectancy number reported above each column due to rounding differences.

Fig 3 shows total life expectancy and life expectancies with and without cognitive impairment at age 50 by gender and prevalence of diabetes, and for the three scenarios. When comparing Figs 2 and 3, it is important to note that life expectancy without cognitive impairment that is shown in Fig 3 will not match the age at first onset of cognitive impairment (Fig 2) for two reasons. First, cognitive impairment is a transient state so that people may recover to normal cognitive functioning (and thus contribute life-years to the not cognitive impaired state). Second, mean age at first onset of cognitive impairment is calculated only for those who experience cognitive impairment while the life expectancies include all individuals.

Remaining life expectancy at age 50 in the Observed scenario is 30.3 years for the entire sample, 27.6 years for men, and 32.1 years for women. Compared to the Observed scenario, a population with no diabetes has approximately a year longer total life expectancy (1.2 years for the entire sample, 1.2 years for men, 1.4 years for women). Approximately 80–85% of this longer life is years without cognitive impairment, but despite diabetes being a strong predictor of cognitive impairment, those without diabetes also have slightly more cognitively impaired years than in the observed scenario.

For individuals who ultimately have diabetes, both the total life expectancy and life expectancy without cognitive impairment are considerably shorter than for those without diabetes. Total life expectancy for men with diabetes is 5.1 years shorter (23.7 vs. 28.8 years) and for women with diabetes 7.3 years shorter (26.2 vs. 33.5 years) than for men and women without diabetes. The majority of these lost years are cognitively healthy years: for men, 4.1 of the 5.1 years, and for women, 6.0 of the 7.3 years. However, men and women with diabetes can expect to spend approximately one year less in cognitively impaired state than individuals without diabetes.

**Fig 4:**
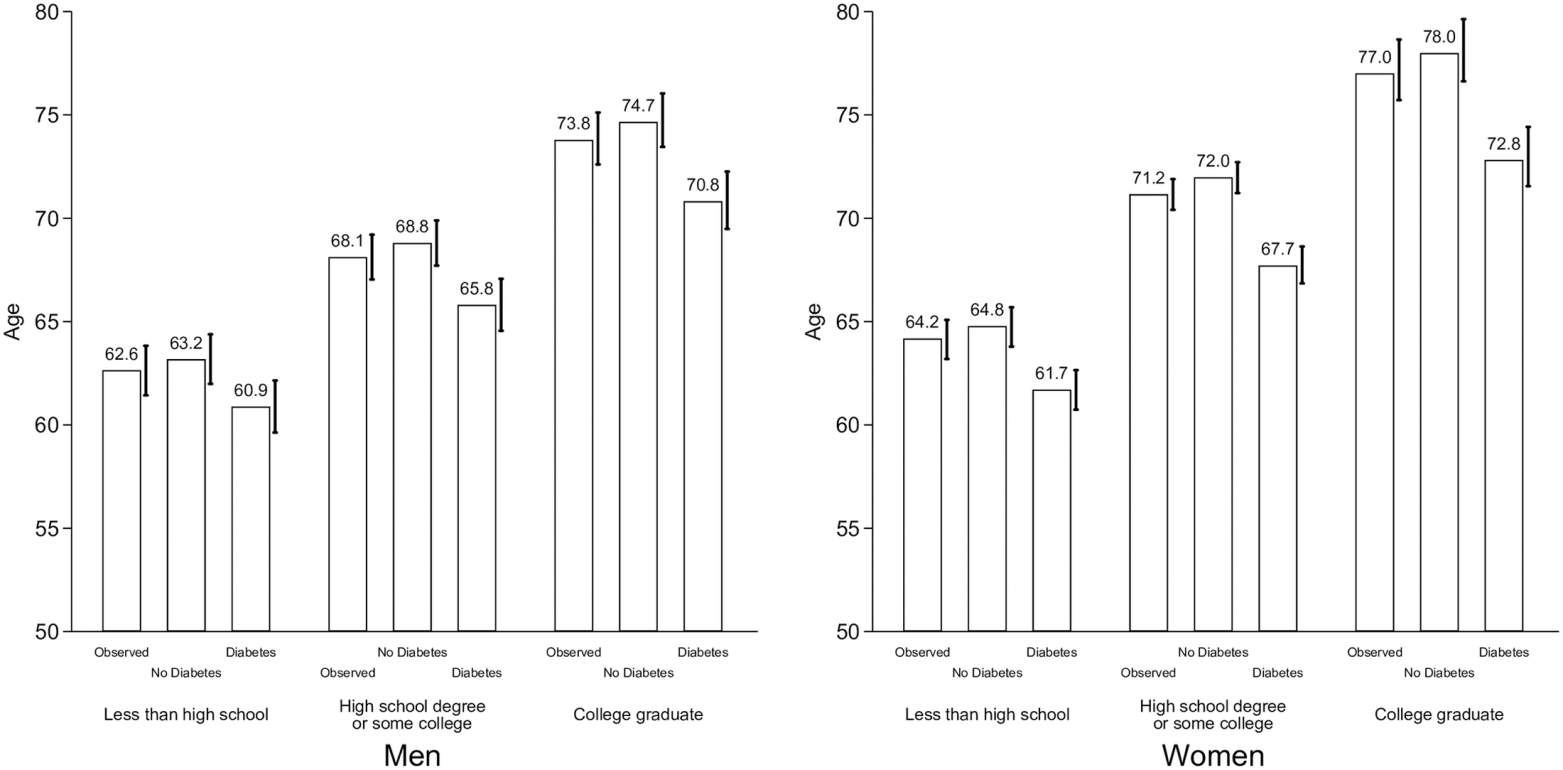
Mean age of first incidence of cognitive impairment by gender, diabetes status, and educational attainment, 2000–2012. **Note**: Authors’ own elaboration with data from the Health and Retirement Study. “Observed” refers to the average prevalence of diabetes. “No Diabetes” assigns zero diabetes to the sample population. “Diabetes” assigns full diabetes to the sample population. The 95% confidence intervals, which are based on 1,000 bootstrap replications, are included on the right of each column.

Fig 4 shows the mean age of first incidence of cognitive impairment by gender, educational attainment, and diabetes status for individuals who are not cognitively impaired at age 50. For women, the mean age at first incidence from non-impaired to impaired cognitive function is 64.2 years for those with less than a high school degree. The mean age increases to 71.2 years for those with a high school degree and to 77.0 years for those with a college degree. Among men, mean ages are slightly lower than those of women. For men with less than a high school degree it was 62.6 years, for those with a high school degree it was 68.1 years, and among college graduates it was 73.8 years. For the No Diabetes scenario, the mean ages at first transition for women are between 0.6 and 1.0 year higher than in the Observed scenario at each of the educational levels. For men, the mean ages at first transition are also higher at all educational levels but the increase ranges between 0.6 and 0.9 years at each of the educational level. Compared to the No Diabetes scenario, in the full Diabetes scenario the mean age at first transition for women is 3.1, 4.3, and 5.2 years lower for those with less than a high school degree, those with a high school degree, and those with a college degree, respectively. Similarly, for men, the mean age at first transition is 2.3, 3.0, and 3.9 years lower for the educational attainment categories mentioned above.

**Fig 5:**
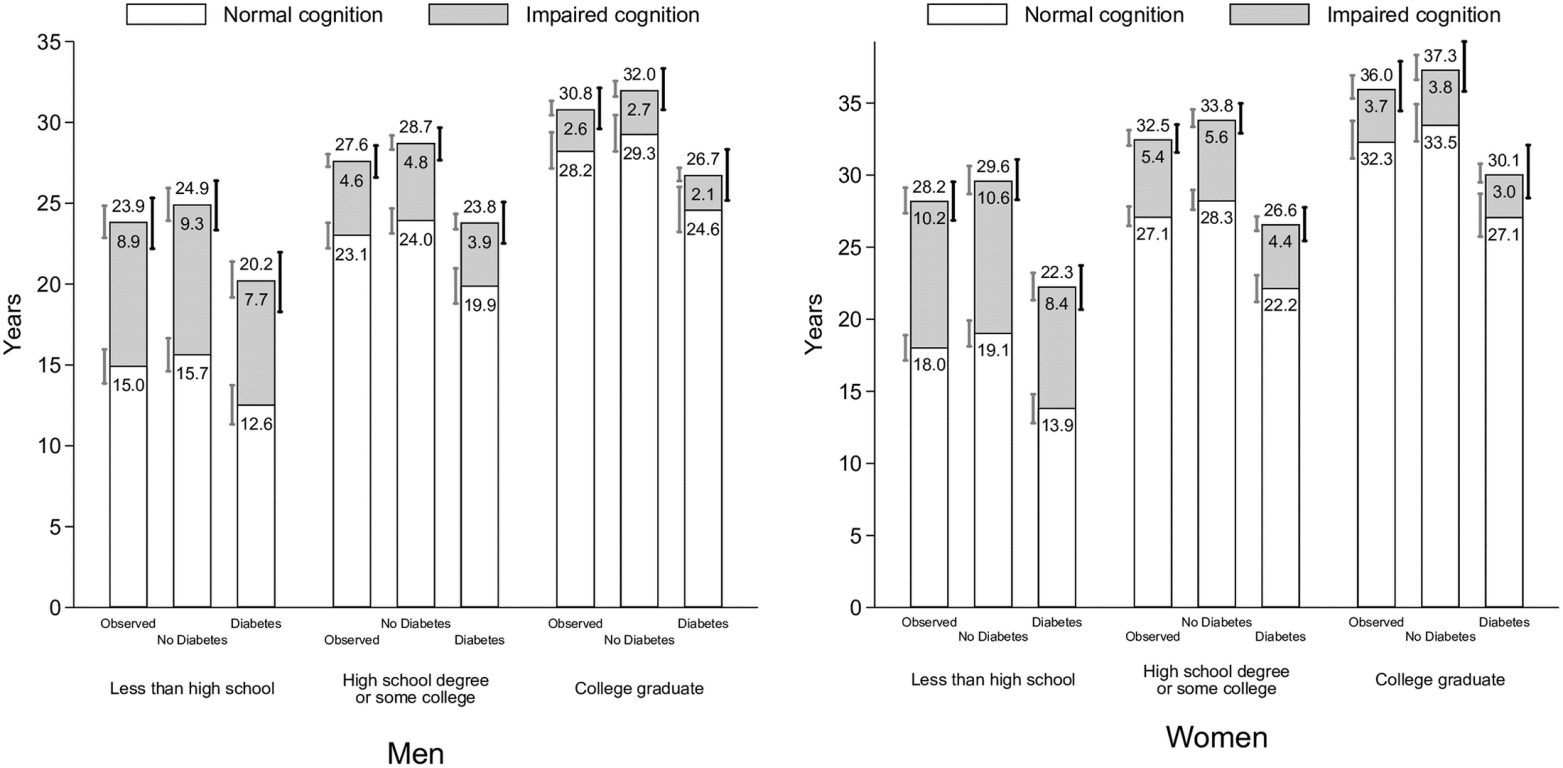
Cognitive impaired and cognitive-impaired free life expectancies at age 50 for older adults in the U.S. by gender, educational attainment, and prevalence of diabetes. **Note**: Authors’ own elaboration with data from the Health and Retirement Study. “Observed” refers to the average prevalence of diabetes. “No diabetes” assigns zero diabetes to the sample population. “Diabetes” assigns full diabetes to the sample population. The 95% confidence intervals on the left of each column (gray) refer to the healthy life expectancy (normal cognition) and to the cognitively-impaired life expectancy (impaired cognition). The 95% confidence intervals, which are based on 1,000 bootstrap replications, on the right of each column (black) refer to the total life expectancy. Both non-impaired and cognitively-impaired life expectancies might not add up to the total life expectancy number reported above each column due to rounding differences.

Fig 5 shows total life expectancy and life expectancies with and without cognitive impairment at age 50 by gender, educational attainment, and prevalence of diabetes for the three scenarios. For women, remaining life expectancy at age 50 in the observed scenario is 28.2 years for those with less than a high school degree, 32.5 years for those with a high school degree, and 36.0 years for those with a college degree. In the No Diabetes scenario, women with a college degree have 3.5 more years of total life expectancy than women with a high school degree and 7.7 more years than women with less than a high school degree. These differences are somewhat similar in the full Diabetes scenario (3.5 and 7.8 more years respectively).

For men, remaining life expectancy at age 50 in the observed scenario is 23.9 years for those with less than a high school degree, 27.6 years for those with a high school degree, and 30.8 years for those with a college degree. In the No Diabetes scenario, men with a college degree have 3.3 more years of total life expectancy than men with a high school degree and 7.1 more years than men with less than a high school degree. These differences are somewhat similar in the full Diabetes scenario (2.9 and 6.5 more years respectively).

## Discussion

Diabetes is a strong predictor of both increased mortality and cognitive decline, and an increasingly prevalent chronic condition among middle- and older-aged Americans. Using a nationally representative sample, we analyzed how diabetes is associated with the onset of cognitive impairment, with total life expectancy, and with life expectancy with and without cognitive impairment at age 50. Four key findings help to understand the dynamics between diabetes and cognitive functioning.

First, diabetes is associated with an earlier onset of cognitive decline and shorter life expectancy with healthy cognitive functioning. Those with diabetes experience, on average, a first incidence of cognitive impairment 3–4 years younger than those without diabetes. Being in a diabetic state from age 50 is associated with a 4–6 years shorter lifetime spent in a cognitively healthy state compared to being in a non-diabetic state.

Second, diabetes is associated with mortality but the majority of the shortened life when compared to those without diabetes is cognitively healthy years. Diabetes is associated with the loss of both cognitively healthy and unhealthy life years, so that total life expectancy at age 50 is approximately five years shorter among men and seven years shorter among women than for those without diabetes. Among both men and women, more than 80% of the lost years are attributable to cognitively healthy years.

Third, despite the strong association between diabetes and cognitive decline, those with diabetes have approximately one year less of cognitively impaired life expectancy at age 50 than those without diabetes. This is because individuals with diabetes have a higher mortality and are therefore less likely to reach advanced ages where the age-related cognitive impairment, independently of diabetes status, becomes a major risk for cognitive impairment.

Fourth, differences in the years of cognitively impaired life expectancy at age 50 between respondents with and without diabetes, become narrower as the educational attainment increases. For women, it goes from 2.2 less years for less than a high school degree, to 1.2 less years for a high school degree, and to 0.8 less years for college graduates. For men, these differences also become narrower going from 1.6 less years for less than a high school degree, to 0.9 less years for a high school degree, to 0.6 less years for a college degree. Our findings confirm results from previous literature that have identified an inverse relationship between diabetes and educational attainment [24].

Our analysis is based on a high-quality longitudinal dataset that is representative of middle-aged and older Americans. We estimate age at onset of cognitive decline and cognitively healthy and impaired life expectancies from transitions between the states, avoiding the bias that is associated with the prevalence based methods such as the Sullivan method [25]. We use the newly developed Markov chain matrix population approach, which avoids the time-consuming simulation step that is necessary in other transition based methods for calculating age at onset of a condition. The estimated total life expectancy at age 50 in our models is 29.7 years for men and women combined for the observation window 2000–2012. This around 0.4–0.5 less years than what the Centers for Disease Control and Prevention (CDC) [26] and the Human Mortality Database (HMD) report for the period 2000–2010 [27], suggesting that the data reflects well the mortality levels in the population.

Our diabetes measurement is based on self-reported answers to the question of ever being diagnosed with diabetes by a physician. Over a quarter of the diabetic population in the U.S. (8 out of 29 million) are thought to be undiagnosed [4]. Therefore, it is likely that we are underestimating the diabetes prevalence by not including undiagnosed cases [28]. For the key results of this paper that reflect the differences in cognitively healthy and impaired life expectancy by diabetes status this means that we may be underestimating the true differences, as our sample without diabetes may in fact contain respondents who have diabetes. Nonetheless, by focusing on the diagnosed diabetes population we provide estimates for a population that has already been identified by the healthcare system and many in this group are likely receiving treatment for their diabetes.

Diabetes type-1 and type-2 affect cognitive impairment differently as may impact non-overlapping cognitive areas [29]. Additionally, diabetes in older adults is divided into long-standing diabetes and elderly-onset diabetes, but we are unable to identify any specific type of diabetes in our findings. Further research should also present results stratified by race/ethnicity in order to gain a deeper perspective on the impact of diabetes and cognitive impairment on life expectancy of older adults. Finally, the measurement of cognitive impairment used in the Health and Retirement Study (HRS) has not been validated to provide a clinical assessment of dementia or to be used as a screen for dementia so our results need to be interpreted with caution regarding the severity of cognitive impairment in the sample.

Our scenarios present results for three different populations: the observed population, a population in which diabetes prevalence is zero, and a population in which everyone has diabetes before death. In this last scenario, the distribution of diabetes diagnoses over age reflects the observed age distribution among those with diabetes, and at each age the transition rates between cognitively healthy and impaired states are calculated for a population that by that specific age has been diagnosed with diabetes. Therefore, the transition probabilities at, for example, age 65 are transitions for a population that has been diagnosed with diabetes at any age before 65. Cognition may decline with time since diagnosis, so our results are likely to underestimate the cognitive burden of diabetes when compared to a hypothetical population in which everyone has diabetes starting from an earlier age, for example, 50.

Our results show that even though individuals with diabetes experience cognitive decline at a younger age than those without diabetes, the former have shorter life expectancy with cognitive impairment. This may be due to two differential mortality effects. First, those with diabetes have an overall elevated mortality, which reduces the likelihood of reaching advanced ages in which the risk of cognitive impairment is high. Second, diabetes and cognitive impairment may be synergistic in their effects on mortality–that is the effect of each condition on mortality may be enhanced when the other is present. Poor management of diabetes among those with cognitive impairment could produce such synergy [30].

The cost of diabetes on length of life is mostly due to reductions in years of cognitively normal functioning. Overall life expectancy is increasing among diabetics, although potentially at a slower rate than in the general population [31, 32]. In addition, the age-specific incidence and prevalence of cognitive impairment appears to be on the decline among older Americans [33]. Further study is needed to ascertain whether those with diabetes are also experiencing declines in cognitive impairment over time. If the welcome progress against mortality among the population with diabetes is not coupled with improved cognition, the cognition burden associated with diabetes may grow over time.
